# Pseudoabsence Generation Strategies for Species Distribution Models

**DOI:** 10.1371/journal.pone.0044486

**Published:** 2012-08-31

**Authors:** Brice B. Hanberry, Hong S. He, Brian J. Palik

**Affiliations:** 1 Department of Forestry, University of Missouri, Columbia, Missouri, United States of America; 2 USDA Forest Service, Northern Research Station, Grand Rapids, Minnesota, United States of America; Vrije Universiteit, The Netherlands

## Abstract

**Background:**

Species distribution models require selection of species, study extent and spatial unit, statistical methods, variables, and assessment metrics. If absence data are not available, another important consideration is pseudoabsence generation. Different strategies for pseudoabsence generation can produce varying spatial representation of species.

**Methodology:**

We considered model outcomes from four different strategies for generating pseudoabsences. We generating pseudoabsences randomly by 1) selection from the entire study extent, 2) a two-step process of selection first from the entire study extent, followed by selection for pseudoabsences from areas with predicted probability <25%, 3) selection from plots surveyed without detection of species presence, 4) a two-step process of selection first for pseudoabsences from plots surveyed without detection of species presence, followed by selection for pseudoabsences from the areas with predicted probability <25%. We used Random Forests as our statistical method and sixteen predictor variables to model tree species with at least 150 records from Forest Inventory and Analysis surveys in the Laurentian Mixed Forest province of Minnesota.

**Conclusions:**

Pseudoabsence generation strategy completely affected the area predicted as present for species distribution models and may be one of the most influential determinants of models. All the pseudoabsence strategies produced mean AUC values of at least 0.87. More importantly than accuracy metrics, the two-step strategies over-predicted species presence, due to too much environmental distance between the pseudoabsences and recorded presences, whereas models based on random pseudoabsences under-predicted species presence, due to too little environmental distance between the pseudoabsences and recorded presences. Models using pseudoabsences from surveyed plots produced a balance between areas with high and low predicted probabilities and the strongest relationship between density and area with predicted probabilities ≥75%. Because of imperfect accuracy assessment, the best assessment currently may be evaluation of whether the species has been sufficiently but not excessively predicted to occur.

## Introduction

Species distribution models provide spatial maps of species ranges and identify influential environmental factors through model selection of predictor variables. Among other uses, this information can be applied to examine effects of land use, climate change, and biotic interactions [1]. Most statistical methods for species distribution models either require presence and absence data or else models may perform better with presence and absence data [2]–[4]. However, even the best surveys only can confirm presence definitively, because a complete census cannot incorporate all variation in time and space [5]. The matter is further complicated by locations that have favorable conditions but are without species presence due to land use, (lack of) natural disturbance, competition, or barriers to dispersal, processes which are difficult to quantify into a model.

Pseudoabsences are surrogates for true absences when true absences are unknown. There are different strategies for generating pseudoabsences [3]–[5]. One common strategy for generating pseudoabsences is simple random selection from within the study area (i.e., background data), without applying limitations based on information known from species presence [3], [6]. Other strategies use information about species presence to guide pseudoabsence selection [7], [8]. These strategies employ knowledge about either 1) spatial distance, by using areas outside where the species is present, either at a buffered distance or from outside the known range, 2) environmental distance, by using lower suitability locations, as determined by the aid of either literature, expert opinion, or presence-only models, or 3) surveyed absence, that is, surveyed plots without a recorded species presence but perhaps not true absences [3]–[5], [7]–[10]. A modification for pseudoabsence generation is a two-step modeling process, first using randomly selected pseudoabsences followed by use of predicted results to select pseudoabsences [6]. Alternative and more intensive approaches exist, including for example, development of expectation-maximization algorithms and detection probabilities [3], [5].

Research has demonstrated the robustness of models with randomly selected pseudoabsences compared to models that use constraints on pseudoabsence generation [6]. Nonetheless, depending on the unknown proportion of the pseudoabsence sample that contains locations with true absences, models based on pseudoabsences generated at random should produce inconsistent results. It seems that any information that can help guide the statistical method to improved predictions is preferable than providing no information. Thus, modeling species distributions using pseudoabsences generated at random followed by selection for low probability areas as absences should provide environmental distance between sites with and without the species. Sites additionally that have been surveyed without detection of the species at least suggest a lower probability of presence than unsurveyed sites. Although this strategy for pseudoabsence generation seems reasonable, it generally has not been used [4].

Modelers need to be aware of potential outcomes that can result due to choice of pseudoabsence strategy. In part because there have not been many comparisons among strategies, results are variable and in disagreement. Mateo et al. [4] compared “target-group” absences (i.e., absences from surveyed plots) to random pseudoabsences and found generally greater accuracy of the target-group absences. In contrast, Lütolf et al. [11] determined that models based on random pseudoabsences were more accurate than models based on pseudoabsences from surveyed sites without the species. One common method of pseudoabsence generation is through profile models, such as ecological niche factor analysis, to determine probable absences. Wisz and Guisan [8] found this strategy to be less accurate than random pseudoabsences and conversely, Engler et al. [12] found this strategy to be more accurate than random pseudoabsences.

Therefore, we compared some of the current strategies for pseudoabsence generation to examine their impacts on models. We modeled tree species distributions using pseudoabsences randomly selected 1) from the entire study area, 2) from plots surveyed without detection of species presence, 3) a two-step process (mimicking profile models) by selecting first from generated pseudoabsences, followed by selection for pseudoabsences from the areas with low predicted probability, 4) a two-step process selecting first from pseudoabsences from the plots surveyed without detection of species presence, followed by selection for pseudoabsences from the areas with low predicted probability. We used Random Forests, an ensemble classifier, as our statistical method and relevant environmental variables to model predictive surfaces for the most common tree species from FIA tree surveys (2004–2008) in the Laurentian Mixed Forest province of Minnesota. Our study will contribute to the discussion of pseudoabsence generation strategies.

## Methods

### Study Area

**Figure 1:**
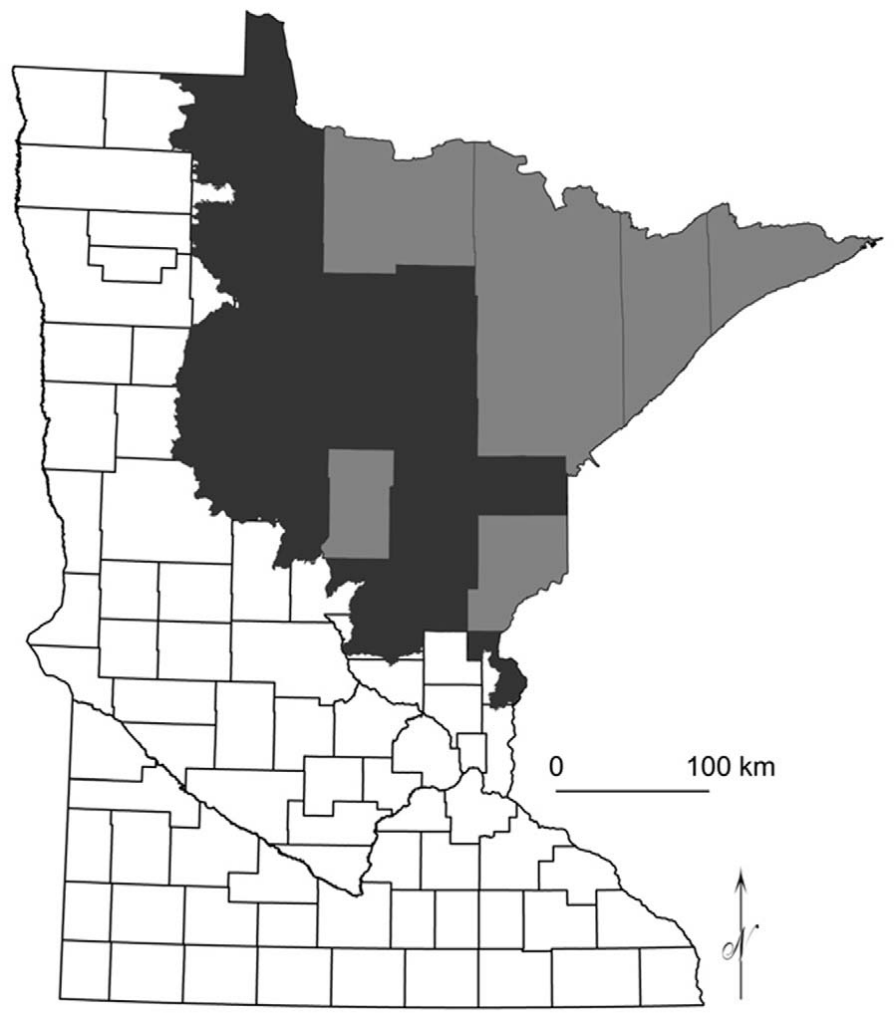
Study area with soils surveys (in black) in the Laurentian Mixed Forest (black and grey) of Minnesota.

The study extent includes about 4.9 million ha of the Laurentian Mixed Forest province of Minnesota, which covers about 9.4 million ha (Figure 1). The Laurentian Mixed Forest has a cool temperate climate, with precipitation increasing from west to east and with temperature increasing from south to north. Elevation ranges from 200 to 700 m. The Laurentian Mixed Forest contains wetlands intermixed with sandy soils on till plains and moraines. The dominant species (trees over 7.6 cm in diameter) of the Laurentian Mixed Forest is quaking aspen (*Populus tremuloides*), which makes up about 20% of forest composition. Black spruce (*Picea mariana)*, northern white cedar (*Thuja occidentalis*), balsam fir (*Abies balsamea*), tamarack (*Larix laricina*), paper birch (*Betula papyrifera*), and black ash (*Fraxinus nigra*) each contribute 6 to 11% of forest composition.

### Spatial Units (Grain) and Environmental Variables

Our spatial units were Soil Survey Geographic (SSURGO) Database (Natural Resources Conservation Service, http://soildatamart.nrcs.usda.gov) polygons, attributes of which are grouped by map units (polygons with similar soil characteristics in a county). We removed water and miscellaneous areas disturbed by human development (e.g., mines, pits, dumps). Also, soils surveys have not been completed in seven counties: Cook, Crow Wing, Isanti, Koochiching, Lake, Pine, and part of St. Louis. After processing, there were about 310,000 polygons with a mean polygon area of 16 ha (SD = 92).

**Table 1:** Environmental variables for modeling.

Type	Predictor
Edaphic	drainage class
	hydric soil presence
	water holding capacity
	pH
	% organic matter
	% clay
	% sand
Topographic	elevation
	slope
	aspect
	solar radiation
	topographic roughness
	wetness convergence
	topographic position
Spatial location based on ecological grouping	subsection
Substrate	bedrock geology

We used sixteen predictor variables that characterize tree occurrence (Table 1). Seven variables were from the soil tables by map unit for each county (i.e., polygons with similar soil characteristics in a county). Categorical soil variables were 1) drainage class (very poorly drained to excessively drained) and 2) hydric soil presence class. For map units with more than one soil component (soil series), we used the categorical variable from the dominant component. We determined depth (cm) to either the bottom of the soil profile or to a soil restriction. We then calculated five continuous soil variables, 1) mean water holding capacity (cm/cm), 2) pH, 3) organic matter (%), 4) clay (%), and 5) sand (%) to the depth, and weighted values by component percentage. From a 30 m DEM (digital elevation model), we calculated seven continuous terrain variables: 1) elevation (m), 2) slope (%), 3) transformed aspect (1+sin(aspect/180*3.14+0.79); [13]), 4) solar radiation (0700 to1900 in 4 hour intervals on summer solstice for re-sampled 60 m DEM), 5) topographic roughness [14], 6) wetness convergence (T. Dilts, http://arcscripts.esri.com), and 7) topographic position index. We calculated the mean value for each topographic variable by a zone (mean area of 210 ha) of soil map unit, land type association (an ecological classification), and bedrock geology, which contained spatially distinct soil polygons. We also joined two more categorical variables to each spatial unit: 1) subsection, which is an ecological subdivision of continuous areas within larger ecological provinces [15] and there are 12 subsections within the study area, and 2) bedrock geology.

**Table 2:** The AUC values using reserved polygons without present cases as pseudoabsences, for models with random pseudoabsences (rand), random pseudoabsences followed by pseudoabsences with probabilities <25% (rand_2), pseudoabsences from surveyed plots (surv), surveyed pseudoabsences followed by pseudoabsences with probabilities <25% (surv_2).

	AUC			
Species	rand	rand_2	surv	surv_2
ashes	0.96	0.81	0.86	0.77
aspens	0.93	0.78	0.80	0.84
balsam fir	0.94	0.75	0.95	0.69
basswood	0.98	0.90	0.96	0.89
birch	0.93	0.78	0.98	0.80
elms	0.89	0.77	0.95	0.84
jack pine	0.99	0.97	0.99	0.97
maples	0.97	0.89	0.96	0.89
red oaks	0.96	0.89	0.97	0.92
red pine	0.99	0.96	0.98	0.95
spruces	0.97	0.91	0.96	0.91
tamarack	0.98	0.95	0.98	0.94
white cedar	0.99	0.96	0.98	0.96
white oaks	0.95	0.88	0.97	0.90
white pine	0.93	0.88	0.97	0.84
yellow birch	0.92	0.86	0.89	0.76
mean	0.95	0.87	0.95	0.87

### Tree Surveys

The U.S. Forest Service Forest Inventory and Analysis (FIA) surveys fixed plots, consisting of four subplots that are each 7.3 m in radius (i.e., each subplot is 167 m^2^), during a five year cycle. We used FIA plots from the latest complete cycle during 2004–2008. The available FIA plot locations are fuzzed (i.e., location moved) and swapped to protect landowner privacy. For a rough idea of tree location, we used available plots downloaded from FIA DataMart (www.fia.fs.fed.us/tools-data). For modeling and prediction, the USDA Forest Service joined our predictor variables to plots in a table based on accurate spatial locations. There were 3994 plots, containing 94644 trees, which intersected with our spatial units. Although we knew if a tree species was present at a plot, we did not know the exact location (i.e., geographic coordinates, which we did not need for modeling) of the plot or if there were unrecorded species outside of the FIA plot but within our spatial units. No specific permits were required for the described field studies.

**Figure 2:**
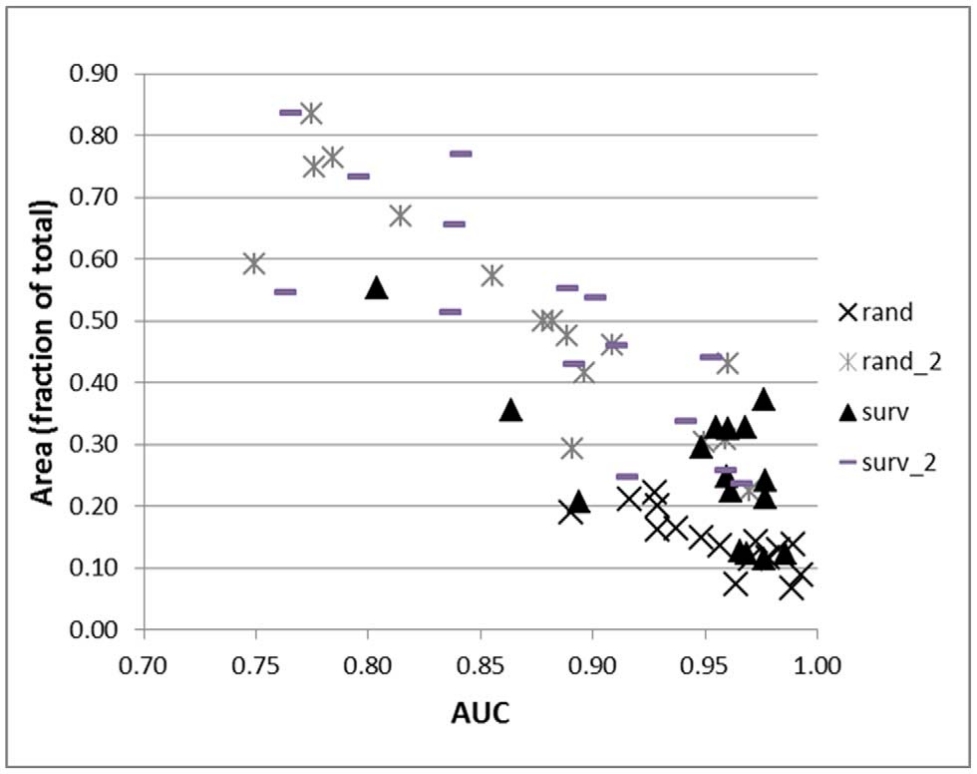
The AUC values compared to area (fraction of total area) predicted as present by species. Random pseudoabsences (rand) had high AUC values and little difference in area by species albeit extremely parsimonious areas, whereas both random pseudoabsences followed by pseudoabsences with probabilities <25% (rand_2) and surveyed pseudoabsences followed by pseudoabsences with probabilities <25% (surv_2) had a range of AUC values and area predicted as present although all predicted areas were large, and pseudoabsences from surveyed plots (surv) had a range of AUC values and area predicted as present.

**Table 3:** Count of FIA surveys (2004–2008), mean predicted probabilities, and area (fraction of total area) of polygons with predicted probabilities ≥75% for models with random pseudoabsences (rand), random pseudoabsences followed by pseudoabsences with probabilities <25% (rand_2), pseudoabsences from surveyed plots (surv), surveyed pseudoabsences followed by pseudoabsences with probabilities <25% (surv_2).

		Mean predicted probability				Area of ≥75% bin			
Species	FIA count	rand	rand_2	surv	surv_2	rand	rand_2	surv	surv_2
ashes	8092	0.40	0.62	0.64	0.90	0.14	0.67	0.35	0.84
aspens	29904	0.45	0.67	0.73	0.88	0.22	0.76	0.55	0.77
balsam fir	7682	0.31	0.45	0.43	0.51	0.16	0.59	0.30	0.55
basswood	2923	0.33	0.49	0.49	0.58	0.12	0.41	0.25	0.43
birch	6419	0.41	0.62	0.62	0.83	0.20	0.75	0.37	0.73
elms	1179	0.54	0.85	0.65	0.82	0.19	0.83	0.33	0.66
jack pine	3147	0.21	0.29	0.27	0.33	0.07	0.22	0.12	0.24
maples	7038	0.35	0.51	0.56	0.69	0.12	0.48	0.32	0.55
red oaks	1917	0.38	0.55	0.54	0.65	0.07	0.29	0.12	0.25
red pine	3219	0.21	0.30	0.29	0.34	0.14	0.43	0.24	0.44
spruces	7525	0.25	0.37	0.35	0.42	0.14	0.46	0.22	0.46
tamarack	5391	0.19	0.26	0.28	0.33	0.13	0.30	0.21	0.34
white cedar	4329	0.15	0.22	0.19	0.23	0.09	0.31	0.11	0.26
white oaks	2716	0.40	0.61	0.60	0.73	0.15	0.50	0.33	0.54
white pine	510	0.41	0.59	0.47	0.65	0.16	0.50	0.13	0.51
yellow birch	186	0.52	0.69	0.57	0.73	0.21	0.57	0.21	0.55
mean		0.35	0.51	0.48	0.60	0.14	0.51	0.26	0.51

We selected the most common overstory trees, about 93,000 trees for 16 species or genus groups. We grouped these species into the following categories: American Basswood (*Tilia Americana*); ashes (*Fraxinus nigra, F. pennsylvanica, F. Americana*); aspens (*Populus tremuloides, P. grandidentata, P. balsamifera*), balsam fir (*Abies balsamea*), birches (*Betula papyrifera, B. cordifolia*), eastern white pine (*Pinus strobes*), elms (*Ulmus americana, U. rubra, U. thomasii*), jack pine (*Pinus banksiana*), maples (*Acer rubrum, A. saccharum, A. saccharinum*), northern white cedar (*Thuja occidentalis*), red oaks (*Quercus nigra, Q. ellipsoidalis, Q. rubra*), red pine (*Pinus resinosa*), spruces (*Picea mariana, P. glauca*), tamarack (*Larix laricina*), white oaks (*Quercus alba, Q. macrocarpa*), and yellow birch (*Betula alleghaniensis*).

**Table 4:** Correlation (all species combined) among predicted probabilities for models with random pseudoabsences (rand), random pseudoabsences followed by pseudoabsences with probabilities <25% (rand_2), pseudoabsences from surveyed plots (surv), surveyed pseudoabsences followed by pseudoabsences with probabilities <25% (surv_2).

	rand	rand_2	surv	surv_2
rand	1.00	0.87	0.69	0.58
rand_2	0.87	1.00	0.67	0.62
surv	0.69	0.67	1.00	0.79
surv_2	0.58	0.62	0.79	1.00

### Pseudoabsence Generation

We generated pseudoabsences using four strategies. We selected from 1) rand, random pseudoabsences from the entire study extent, without exclusion of polygons with tree presence (presence was unknown for the entire extent but known for the polygons that intersected FIA plots), 2) rand_2, a two-step process selecting first from randomly generated pseudoabsences, followed by selection for pseudoabsences from the areas with predicted probability <25%, 3) surv, plots surveyed without detection of species presence, 4) surv_2, a two-step process selecting first for pseudoabsences from the plots surveyed without detection of species presence, followed by selection for pseudoabsences from the areas with predicted probability <25%.

**Figure 3:**
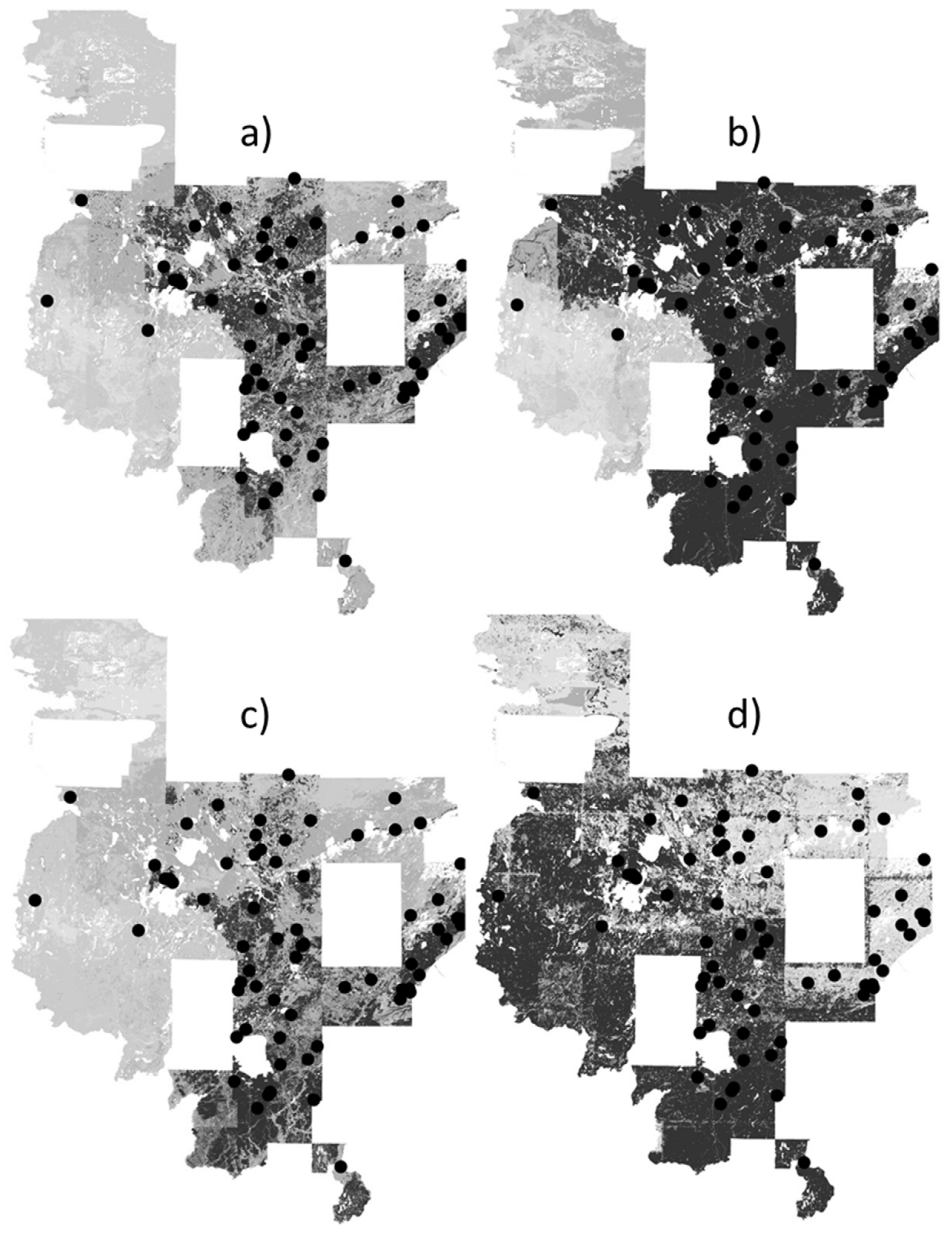
Predicted probabilities (black indicates areas of ≥75% predicted probability) for uncommon yellow birch (black circles indicates approximate location) by models with a) random pseudoabsences, b) random pseudoabsences followed by pseudoabsences with probabilities <25%, c) pseudoabsences from surveyed plots, d) surveyed pseudoabsences followed by pseudoabsences with probabilities <25%.

**Figure 4:**
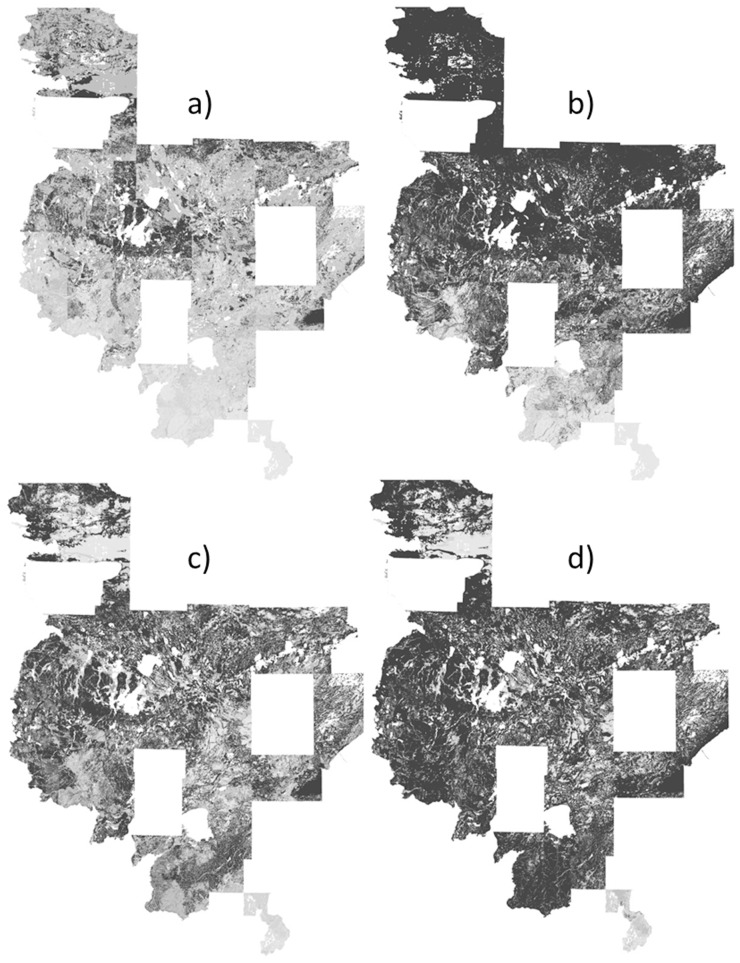
Predicted probabilities (black indicates areas of ≥75% predicted probability) for widespread aspens by models with a) random pseudoabsences, b) random pseudoabsences followed by pseudoabsences with probabilities <25%, c) pseudoabsences from surveyed plots, d) surveyed pseudoabsences followed by pseudoabsences with probabilities <25%.

**Figure 5:**
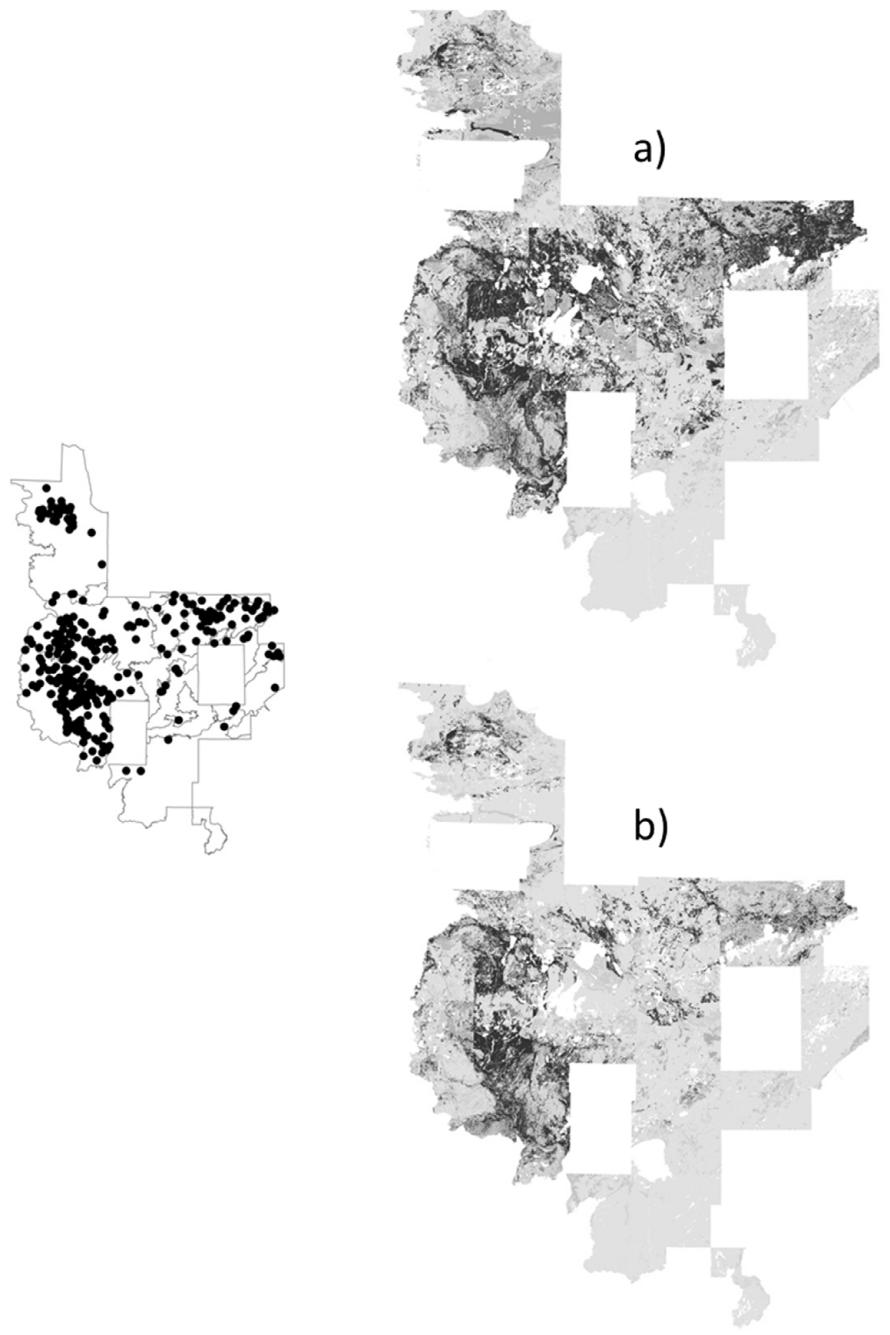
Predicted probabilities (black indicates areas of ≥75% predicted probability) for jack pine by models with a) random pseudoabsences followed by pseudoabsences with probabilities <25% and b) pseudoabsences from surveyed plots.

**Figure 6:**
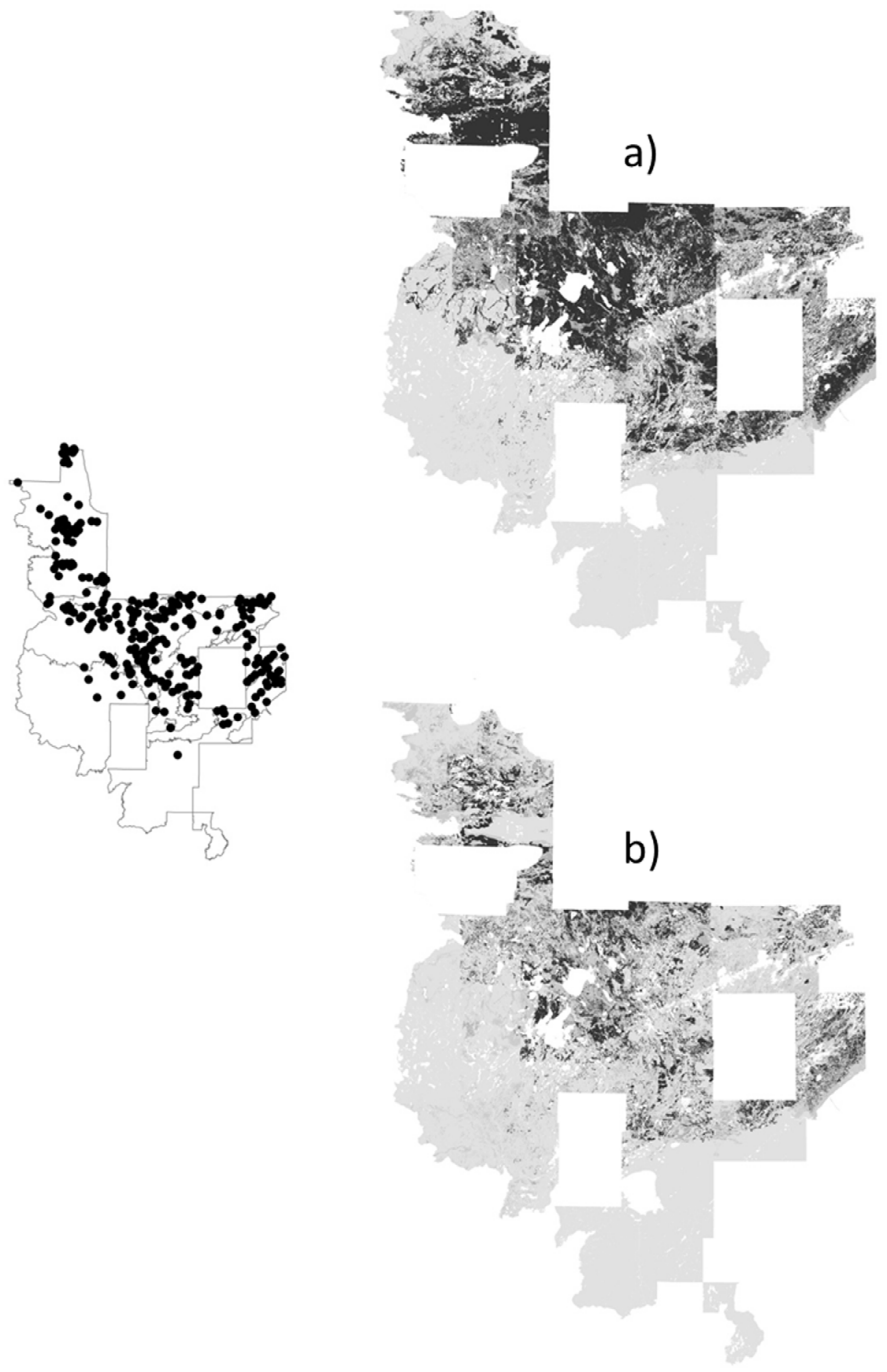
Predicted probabilities (black indicates areas of ≥75% predicted probability) for white cedar by models with a) random pseudoabsences followed by pseudoabsences with probabilities <25% and b) pseudoabsences from surveyed plots.

**Figure 7:**
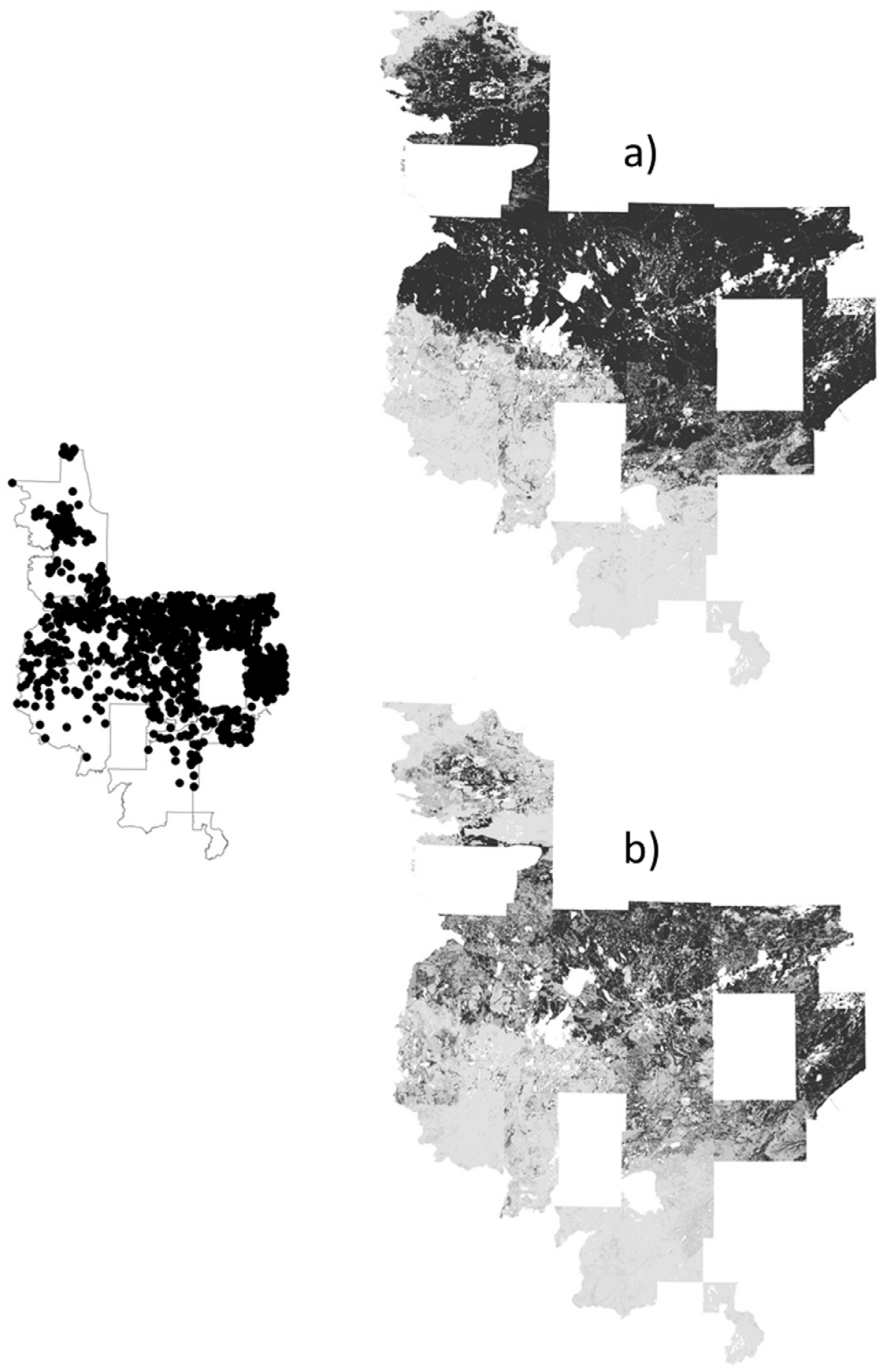
Predicted probabilities (black indicates areas of ≥75% predicted probability) for balsam fir by models with a) random pseudoabsences followed by pseudoabsences with probabilities <25% and b) pseudoabsences from surveyed plots.

For each species or species group, we joined the points to the soil polygons. Due to limits on records for modeling, we then selected a proportionate number of presences relative to the number of pseudoabsences to reduce the inequality between the number of samples with and without the tree species for the modeling dataset. We randomly selected 67% of polygons with the species, up to 2500 polygons, for modeling, and held back the rest for validation. For pseudoabsences, we then randomly selected either 1) 2500 polygons from the entire study extent or 2) 67% of polygons without the species, up to 2500 polygons, for modeling and held back the rest for validation. After modeling, for the two-step processes, we selected 2500 polygons from the areas with low predicted probability after either pseudoabsence generation from 3) the entire study extent or 4) the surveyed plots without present cases.

### Modeling and Prediction

Decision trees are nonparametric options to linear models based on partitioning into classes. Random Forests [16]–[17], improves on decision trees by growing multiple trees grown in parallel and using random subsets of both predictor variables and training data. Classification results from bootstrap aggregation (bagging) by the majority vote of the many trees. We used the randomForest package [18] in R statistical software (R development core team 2010) with the *sampsize* option (which is sampled without replacement), where we set the bag fraction, or subsampling rate, at 67% of the selected polygons with the species. To focus modeling on prediction of presence more than absence, we then specified a modeling prevalence, or ratio of present cases to total cases, of 0.8. We set the number of trees at 1000 and the number of variables randomly sampled at each split as the square root of the number of predictors.

### Validation

We did not rely only on standard assessments. We used the reserved sample with recorded species (33% of the data) and instead of (unknown) true absences, the reserved sample of surveyed sites without recorded species to calculate AUC values for predicted probabilities (ROCR package [19] in R). Because the decision of the threshold dividing predicted presence and absence of species is complicated and unresolved, with many justifiable choices (e.g., [20]–[24]) that can affect comparisons among pseudoabsence generation strategies, we did not use true positive rate and true negative rates. Although there are problems with AUC values as well, the point of all these values is only to show that the models are accurate when comparing predictions with observations. We calculated the mean of predicted probabilities. We grouped the predictions into 4 bins, 0–<25%, ≥25–<50%, ≥50–<75%, ≥75–100%, and designated the 75–100% bin as probable presence (i.e., we established the division between presence and absence of species at a value close to the modeling prevalence [25]). We then determined the area of probable presence as a fraction of the total area.

Although predicted probabilities are for presence, there should be some apparent difference between models for species based on abundance. That is, the most abundant species should not have a distribution that resembles the extent for a relatively rare species. We ran a simple regression (Proc Reg; SAS software, Version 9.1, Cary, North Carolina, USA) to look for a relationship between the count of the tree species and either the mean predicted probability or area of predicted presence. We correlated predicted probabilities among the pseudoabsence generation strategies by species and determined statistical difference using Kruskal-Wallis and ANOVA (Proc npar1way) tests. Lastly, we mapped and visually compared the distributions.

## Results

All the strategies were accurate and similar in accuracy. Mean AUC values using surveyed pseudoabsences were slightly lower for the two-step strategies than the one-step strategies. Mean AUC values were 0.87 for both two-step strategies compared to 0.95 for both one-step strategies using the reserved polygons without present cases as the pseudoabsences (Table 2).

Pseudoabsence generation completely affected the probability of occurrence and the area (fraction of total area) that species were predicted as present, designated by the ≥75% bin (Table 3, Figure 2). The random pseudoabsence strategy performed well considering the parsimonious predictions; mean predicted probability was 0.35 (range of 0.15 to 0.54) and mean ≥75% bin area was 0.14 (with a narrow range of 0.07 to 0.22). The surveyed pseudoabsence strategy had a mean predicted probability of 0.48 (range of 0.19 to 0.73) and a mean ≥75% bin area of 0.26 (range of 0.11 to 0.55). The two-step strategy with a random pseudoabsence first step had an overall mean predicted probability of 0.51 (range of 0.22 to 0.85) and for the ≥75% bin, a mean bin area of 0.51 (range of 0.22 to 0.83). The two-step strategy with a surveyed pseudoabsence first step had a mean predicted probability of 0.60 (range of 0.23 to 0.90) and a mean ≥75% bin area of 0.51 (range of 0.24 to 0.84). Compared to the ≥75% bin area of the two-step pseudoabsence strategies, the random pseudoabsence strategy produced about 28% of the area and the surveyed pseudoabsence strategy had about 52% of the area.

The original selection of pseudoabsence generation, either randomly or by survey, preserved the greatest correlation among strategies (*r* = 0.87 for the one-step and two-step random pseudoabsence strategies and *r* = 0.79 for the one-step and two-step surveyed pseudoabsence strategies; Table 4). All other correlations between strategies of different pseudoabsence strategies had correlation values that ranged from 0.58 to 0.69. Correlation *p* values were <0.0001 for all species. Kruskal-Wallis and ANOVA (Proc npar1way) tests to compare predicted probabilities by species also showed differences were significant among pseudoabsence generation strategies for all species (*p* values were <0.0001 for all species).

We determined some discrimination for absence through a relationship between density (count of each species for the survey area) and mean predicted probability and area for the 75–100% predicted probability bin. Of course, there are species with restricted ranges and greater density within the range, such as white cedar, which had a moderate abundance but the lowest predicted probabilities and counts for the 75–100% predicted probability bin. The *R* ^2^ values for a polynomial regression (*y* = *a*+*b*_1_X+*b*_2_X^2^) between count and 1) area predicted as present and 2) predicted probabilities were 0.86 and 0.61, respectively, for the one-step strategy based on surveyed pseudoabsences. The *R* ^2^ values were ≤0.30 for the other three pseudoabsence strategies.

One main product of species distribution modeling is the species distribution map, which provides an important assessment. The distribution maps showed over-prediction (relative to the known frequency in the landscape) for uncommon yellow birch by the two-step strategies, particularly the two-step strategy with a surveyed pseudoabsence first step (Figure 3). Conversely, the random pseudoabsence strategy produced under-prediction for widespread aspens compared to predictions for rarer species (Figure 4). A closer look at the two strategies with moderate predicted probabilities, the surveyed pseudoabsence strategy and the two-step strategy with a random pseudoabsence first step, demonstrated similar patterns between the two strategies, but the surveyed pseudoabsence strategy produced a smaller area of predicted probabilities ≥75% (see jack pine, white cedar, and balsam fir examples for moderate counts of 3000 to 8000 individuals; Figures 5–6 7). Consequently, maps from surveyed pseudoabsence strategies had more variation at finer scales and were more likely to avoid commission (false positive) errors.

## Discussion

Pseudoabsence generation may be one of the most important factors that determine species distribution models, however this may not be apparent in accuracy assessment metrics. There was little practical difference, at least between the one-step strategies, in our accuracy assessment using known presences and surveyed absences, making it important to evaluate differences in values for mean predicted probability and areas of predicted probability bins and examine distribution maps to make sure the coverage was reasonable. Values for mean predicted probability and area in each predicted probability bin varied considerably due to strategies for generating absence. The distribution maps ranged from prediction of species in small areas (i.e., models based on random pseudoabsences) to prediction of species in large areas (the two-step strategies).

Maps based on selecting pseudoabsences from surveyed plots provided a range in area of probable presence depending on species abundance. Models using pseudoabsences from surveyed plots had the strongest relationship between density and both predicted probability and area of polygons with predicted probabilities ≥75%. There was an increase in predicted probabilities and counts for the 75–100% predicted probability bin as presence counts increased from yellow birch (186 recorded individuals) to aspens (29904 recorded individuals). Engler et al. [12] and Hernandez et al. [25] recommended selection of models that are accurate (low omission) and cover the least area (i.e., presumably have the fewest commission errors). Models based on random pseudoabsences certainly were parsimonious in the area of high predicted probability compared to accuracy. Nevertheless, these models also were conservative with little range in area predicted for each species, suggesting errors of omission in the species distribution due to conflicting areas with both presence and absence. Aspen, a widespread and dominant species, was not assigned great predicted probability except in restricted areas, and we did not find that a satisfactory distribution for a species that was at least 3.5 times more common than any other species. Conversely, two-step strategies, based on pseudoabsences from low predicted probabilities, assigned a high probability of presence to areas outside of the narrowly specified environmental conditions of pseudoabsence. Two-step strategies thus produced too much environmental distance between the pseudoabsences and recorded presence of the species, overestimating species presence and increasing commission errors [2].

Although we reached some of the same conclusions as other authors, we developed conclusions based on (predicted area of presence in) distribution maps, one of the end goals of modeling, rather than relying solely on accuracy metrics. In addition to the unknown and variable amount of overlap between random pseudoabsences and true absences, it may be that disagreement about pseudoabsences is caused in part by reliance on imperfect accuracy metrics alone [26]–[29]. Mateo et al. [4], who found greater accuracy of “target-group” absences than random pseudoabsences, used AUC (area under the receiver operating curve, a plot of true positive rate against false positive rate) values. Lütolf et al. [11], who found random pseudoabsences were more accurate than pseudoabsences from surveyed sites without the species, used AUC values, the Kappa statistic, and adjusted *D* ^2^. For presence-only data, AUC values are based on pseudoabsences, which may not represent absences and thus, may confound AUC values. Modeling with the use of predicted absences from profile models may produce similar results to our two-step strategies, namely, over-prediction, due to enhanced separation between pseudoabsences and presences. Wisz and Guisan [8], who found random pseudoabsences more accurate than pseudoabsences from profile models, used AUC values and conversely, Engler et al. [12], who found pseudoabsences from profile models more accurate than random pseudoabsences, used a variety of metrics, including the Kappa statistic and adjusted *D* ^2^.

Absence, or pseudoabsence, generation greatly affects species distribution models. Inclusion of pseudoabsence data with unknown probability of absence can produce poor spatial representation through either under- or overrepresentation of the species. Models based on selecting pseudoabsences from surveyed plots did not restrict either presence (as models based on random pseudoabsences did) or absence (the two-step models) to limited areas. Our use of polygons rather than pixels is unusual; however, using a reduced set of FIA plots (to protect landowner privacy) associated with predictor variable scaled to 90 m grid cells, the difference in predicted area of presence persisted regardless of grain and indeed, the difference was amplified**.** Due to the limitations in current accuracy assessment methods, partial reliance on area and distribution maps to evaluate whether the species has been sufficiently but not excessively predicted to occur may help provide the best assessment.
